# Three-Dimensional Optical Scanning in Post–kala-azar Dermal Leishmaniasis (PKDL)

**DOI:** 10.4269/ajtmh.19-0370

**Published:** 2019-12-30

**Authors:** Eduard E. Zijlstra, Niels Liberton, Ahmed M. Musa, Sjoerd te Slaa, Jan Wolff

**Affiliations:** 1 Drugs for Neglected Diseases Initiative (DNDi), Geneva, Switzerland;; 2 Department of Oral and Maxillofacial Surgery/Oral Pathology and 3D Innovation Lab, VU University Medical Center, Amsterdam, The Netherlands;; 3 Institute of Endemic Diseases, University of Khartoum, Khartoum, Sudan;; 4 Division for Regenerative Orofacial Medicine, Department of Oral and Maxillofacial Surgery, University Hospital Hamburg-Eppendorf, Hamburg, Germany;; 5 Fraunhofer Research Institution for Additive Manufacturing Technologies IAPT, Hamburg, Germany

## Abstract

Post–kala-azar dermal leishmaniasis may occur after successful treatment of visceral leishmaniasis and is characterized by macules, papules, or nodules in the skin, with varying size. The response to antileishmanial therapy remains difficult to assess because there are presently no reliable biomarkers. To date, skin lesions are clinically assessed for decrease in size or change in color, which is invariably subjective. Novel 3-dimensional optical scanning devices offer safe and field-adapted methods to objectively assess skin lesions for changes over time in size and color that can be quantified with great accuracy.

A 23-year-old man presented with a skin rash of 3 months duration. He was from White Nile state, Sudan, an area endemic for visceral leishmaniasis (VL, kala-azar). He had had VL 7 months previously for which he had been treated with sodium stibogluconate, with good results. Three months later, he developed a skin rash mainly on the face and the limbs and had not shown improvement since. On examination, he was well and afebrile; no abnormalities were found on physical examination, particularly there was no hepatosplenomegaly or lymphadenopathy. The patient had developed mainly a papular rash on his face, whereas a predominantly macular rash consisting of irregular spots with hypopigmentation was visible on his limbs. Parasitological examination of a slit skin smear revealed *Leishmania* parasites by microscopy.

To accurately document the size of the lesions and to estimate the volume of the papules, the authors used a handheld 3-dimensional optical scanner (Artec 3D, Luxembourg) for the first time for this indication (Figure 1). Optical scanning of the lesion took approximately 60 seconds. The 3-D scan data were polygonized using Artec Studio v.12 professional software (Artec 3D) and exported as an STL (stereolithography) model into GOM Inspect metrology software (GOM GmbH, Braunschweig, Germany) to calculate the dimensions of the lesions.

**Figure 1.**
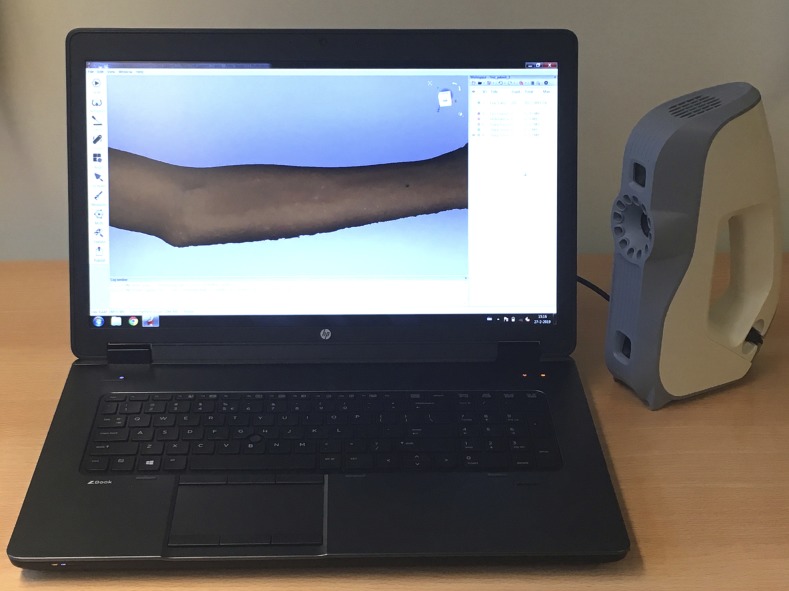
The study setup with the handheld Artec optical scanner and the computer showing the scanned arm. This figure appears in color at www.ajtmh.org.

Four index lesions on the face and two on the arm were randomly selected and the lesion circumferences and volumes were calculated. In addition, the level of elevation of each lesion above the normal skin was measured using a height map (Figures 2 and 3).

**Figure 2.**
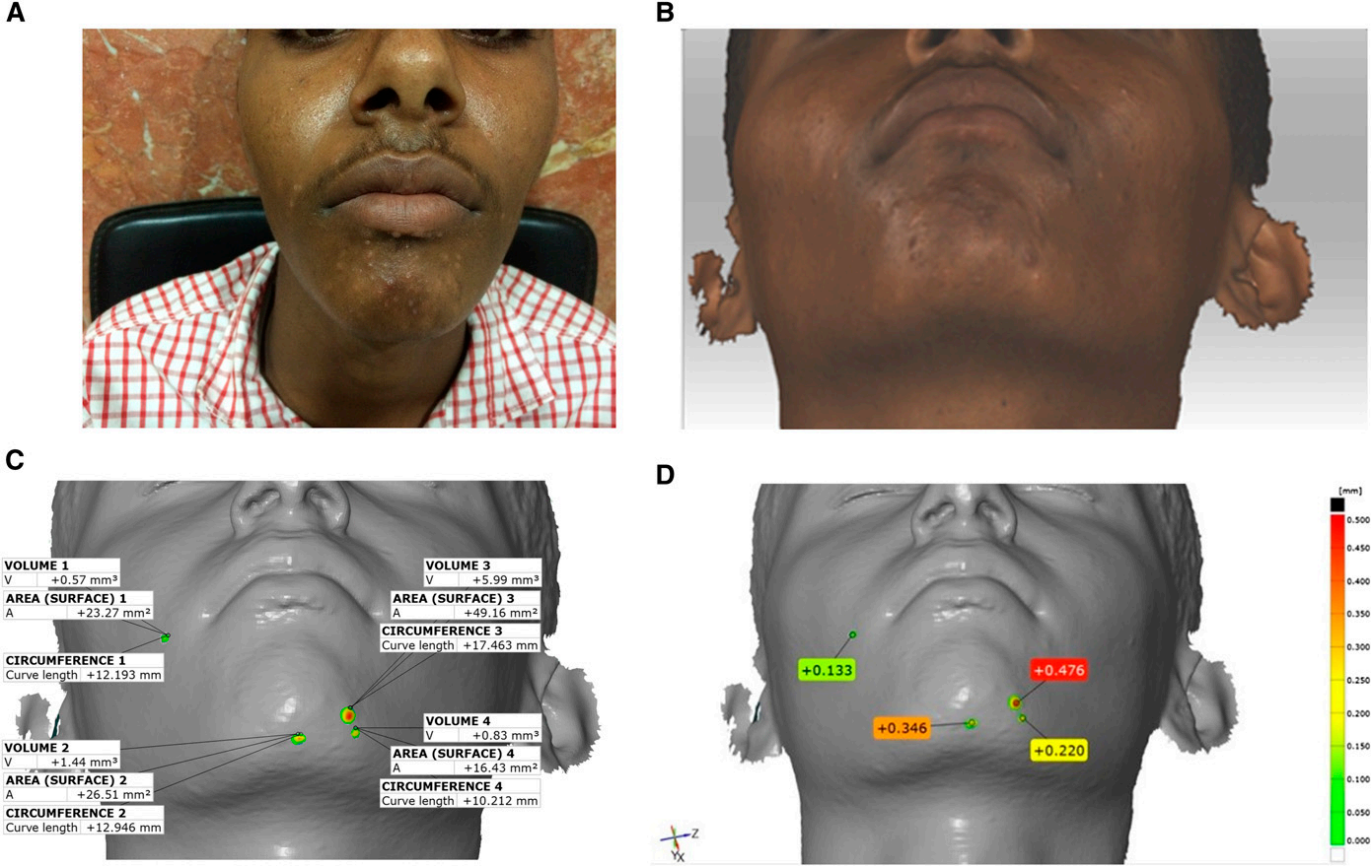
(**A**) Two-dimensional photograph showing multiple papules on the chin and around the mouth. (**B**) Three-dimensional scan of the area of interest, in this case, the chin. (**C**) The image in **B** was exported as an stereolithography model and then imported into GOM Inspect metrology software, allowing the measurement of circumference, surface area, elevation above the level of normal skin, and volume of four randomly selected papules. (**D**) The “heightmap” shows the elevation above the level of the skin using color-coding. The images are reproduced with informed consent from the patient.

**Figure 3.**
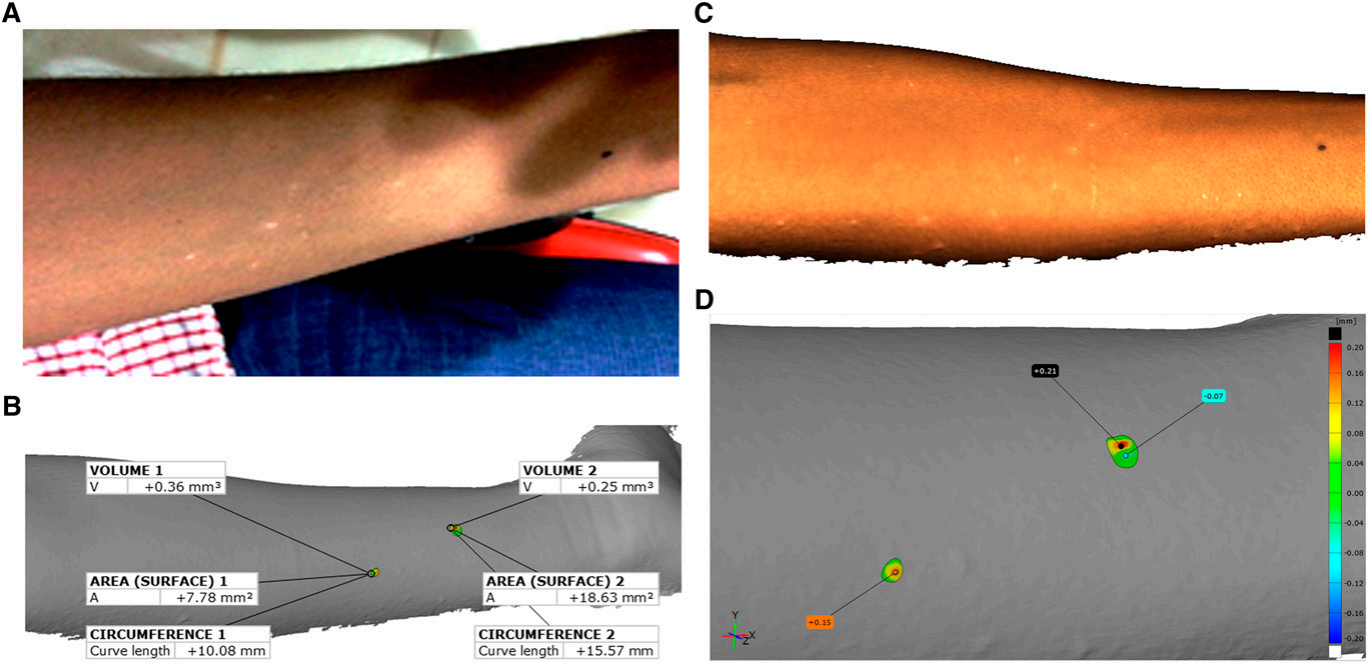
(**A**) Two-dimensional photograph showing multiple macules and papules on the forearm. (**B**) Three-dimensional scan of the area of interest, in this case, the forearm. (**C**) The image in **B** was exported as an STL model and then imported into GOM Inspect metrology software, allowing the measurement of circumference, surface area, elevation above the level of normal skin, and volume of two randomly selected areas. (**D**) The “heightmap” shows the elevation above the level of the skin using color-coding. The images are reproduced with informed consent from the patient.

## DISCUSSION

Post–kala-azar dermal leishmaniasis (PKDL) is a common complication after treatment of VL that occurs in up to 50–60% of cases in Sudan, whereas in in the Indian subcontinent, PKDL occurs in 10–20% of all cases.^1^ Post–kala-azar dermal leishmaniasis patients are usually not ill, apart from the rash. In Asia, the rash occurs within 1–3 years after VL and the macular type is most common; in Africa, papulonodular rash is more common that usually occurs within 6 months after VL; mixed types occur in both areas. Whereas in Africa, spontaneous cure is the rule, all patients are treated in Asia.^1^ Improvement is often assessed clinically, and various methods have been designed that include reduction in grade of severity and distribution and counting the number of lesions in a particular area of the body.^2^ Sequential standardized 2-D photography is currently used to assess skin lesions; however, 2-D images have limited accuracy and reproducibility and their use is subject to error. Macular lesions are notoriously difficult to assess, as they may persist long after clinical cure because repigmentation is slow and changes in color are difficult to record.^3^ As PKDL patients are considered a reservoir of *Leishmania* parasites and may, thus, play a role in the transmission of VL, there is a need for efficacious and safe treatment regimens. Assessment of the response to treatment is important to determine cure, and new accurate methods of assessing skin lesions in PKDL patients are, therefore, sought.

Bearing the aforementioned drawbacks in 2-D photography in mind, in this study, we have explored the usability of 3-dimensional optical scanning in the assessment of PKDL lesions. The handheld scanner is light weight and comes with a durable portable suitcase. It takes approximately 10 minutes to perform an optical scan with minimal discomfort for the patient. The scanner is connected to a laptop or computer which allows immediate interpretation of the lesion, and if necessary, the data can be sent by the Internet to a central location for expert advice. Follow-up scans can be superimposed on the previous scan(s) and aligned using appropriate software to measure differences. For both the scanning and alignment, a short training is required.

For aforementioned reasons, optical 3-D scanners are becoming increasingly popular in clinical settings because they are inexpensive, portable, and easy-to-use, and can create high-resolution 3-D models of virtually any part of the body.^4,5^ Such 3-D scanners typically rely on structured light to capture the geometry of a region of interest. This technology offers the possibility of simultaneously acquiring a series of color images of the skin (“texture”) over time.

This first case shows that 3-D optical scanning for PKDL is promising as it allows objective, quantitative assessment of changes in diameter, circumference, and volume of the lesions over time that can be tested for significance statistically. Further studies are needed to standardize and validate the method in various disease presentations, including macular, papulonodular, or mixed (polymorphic) lesions, at diagnosis and during follow-up. This objective measurement tool is a major improvement to other forms of clinical assessment that are invariably subjective and could be developed as a potential biomarker.

Optical 3-D scanning may also potentially be used for the diagnosis and monitoring of various, particularly, tropical dermatological conditions, including fungal infections (chromoblastomycosis, sporotrichosis, and mycetoma), bacterial infections (tropical ulcer, Buruli ulcer, and leprosy), and parasitic infections (cutaneous leishmaniasis). It also has potential for use in noninfectious conditions such as vitiligo and burn injuries.^6^

## CONCLUSION

Handheld optical 3-D scanners offer an alternative to existing 2-D medical imaging technologies such as medical photography, MRI, and CT scans in developing countries. The technology has the potential to revolutionize the assessment and monitoring of PKDL and other skin lesions in clinical settings, as well as under field conditions.
